# Electronic structures and optical characteristics of fluorescent pyrazinoquinoxaline assemblies and Au interfaces

**DOI:** 10.1038/s41598-021-96437-x

**Published:** 2021-08-20

**Authors:** Soyeong Kwon, Dong Yeun Jeong, Weon-Sik Chae, Kyungju Noh, P. Devi, Luciano Colazzo, Youngmin You, Taeyoung Choi, Dong-Wook Kim

**Affiliations:** 1 Department of Physics, Ewha Womans University, Seoul, 03760 South Korea; 2 Division of Chemical Engineering and Materials Science, and Graduate Program in System Health Science and Engineering, Ewha Womans University, Seoul, 03760 South Korea; 3 Daegu Center, Korea Basic Science Institute (KBSI), Daegu, 41566 South Korea; 4 Center for Quantum Nanoscience, Institute for Basic Science (IBS), Seoul, 03760 South Korea; 5 Ewha Womans University, Seoul, 03760 South Korea

**Keywords:** Materials science, Nanoscience and technology, Physics

## Abstract

Understanding the excitonic processes at the interfaces of fluorescent π-conjugated molecules and metal electrodes is important for both fundamental studies and emerging applications. Adsorption configurations of molecules on metal surfaces significantly affect the physical characteristics of junctions as well as molecules. Here, the electronic structures and optical properties of molecular assemblies/Au interfaces were investigated using scanning probe and photoluminescence microscopy techniques. Scanning tunneling microscopy images and tunneling conductance spectra suggested that the self-assembled molecules were physisorbed on the Au surface. Visible-range photoluminescence studies showed that Au thin films modified the emission spectra and reduced the lifetime of excitons. Surface potential maps, obtained by Kelvin probe force microscopy, could visualize electron transfer from the molecules to Au under illumination, which could explain the decreased lifetime of excitons at the molecule/Au interface.

## Introduction

The transport and annihilation of excitons along assemblies of π-conjugated molecules are the key processes that govern the performance of their electronic and optoelectronic devices^1–5^. Heteromolecular excitonic processes are important for the generation of interfacial charge carriers since most devices are based on thin-film heterojunctions. In particular, there have been intensive research efforts to understand the excitonic processes at organic/metal interfaces for fundamental studies as well as practical device applications^6–18^. Alignment of the Fermi level of electrodes with respect to the energy levels of semiconductor materials determines the interfacial energy barriers of charge injection, which is critical to improve the quantum efficiency of light emitting devices^6–9^. Charge-selective (either electrons or holes) contacts enable dissociation of the photogenerated excitons and promote the collection of charge, leading to the large open-circuit voltage of photovoltaic devices^19,20^.

In inorganic semiconductors, charge carriers can be described by delocalized waves, and the electrical conduction can be explained by the energy band model. Transport measurement and analysis techniques have been well established to investigate the energy band alignment at metal/inorganic-semiconductor interfaces^21^. However, these methods cannot be applied to metal/organic-semiconductor junctions due to their distinct conduction mechanisms. Transport in organic semiconductors takes place by thermally activated hopping between localized states since weak intermolecular interactions are inherent in these materials^8,22^. In addition to transport measurements, scanning tunneling microscopy (STM) characterizations are useful to study metal/semiconductor junctions^13–18^. STM studies can directly visualize the intra- and intermolecular structures at the metal/semiconductor interface with an atomic-scale spatial resolution^11–15^. Adsorption configurations on various metal surfaces and self-assembled structures of molecules play crucial roles in determining the electronic properties of the junctions and physical characteristics of molecules^6–18^. Chemical reaction at the organic/metal interfaces can form gap states and pin the Fermi level, which can modify the interfacial electronic structures^6–9^. Scanning tunneling spectroscopy (STS) measurements can visualize the electronic states of molecules on metal substrates^14–18^.

Here, the electronic structures and optical properties of supramolecule/Au interfaces were investigated using scanning probe and photoluminescence microscopy techniques. Au is one of the most popular electrode materials for both transport measurements and device fabrication^6–18^. DY1 was employed because of its excellent thermal, photochemical, and electrochemical stabilities were established in our previous studies^5^. DY1 is based on a planar pyrazinoquinoxaline core that exerts a strong intermolecular adhesive force due to π–π interactions. These intermolecular interactions would be beneficial for the formation of supramolecular self-assemblies of DY1. It was also anticipated that the highly symmetrical molecular structure would facilitate the formation of two-dimensional reticular assemblies of DY1 onto crystalline substrates. The propensity for intermolecular packing and lateral layering would provide a valuable opportunity to investigate molecular excitons at DY1-based solid-state heterojunctions. This work can provide us with important clues to answer the following questions: (1) is there any structural modification in the molecular self-assemblies of DY1 by interactions with Au, thereby leading to variations in the molecular energy levels of DY1? (2) Does charge transfer occur at the DY1/Au interface? (3) Do we need to consider chemical reactions and resulting defect formation at the DY1/Au interface?

## Results and discussion

The molecular construct of DY1 involves pyrazinoquinoxaline with four phenyl groups at the 2, 3, 7, and 8 positions. The phenyl substituents are predicted (B3LYP/6-311+G(d,p)) to be virtually vertical to the pyrazinoquinoxaline core (“Supporting Information (SI)”), Table S1). The lowest-energy singlet excited state involves electronic transition between the highest occupied molecular orbital (HOMO) and the lowest unoccupied molecular orbital (LUMO) localized at pyrazinoquinoxaline (“SI”, Table S2 and Fig. S1). DY1 is expected to display weak fluorescence emission due to intersystem crossing to the triplet state, as inferred from the presence of the closely lying triplet n–π* transition state. Indeed, DY1 exhibits a photoluminescence (PL) emission with a peak wavelength (*λ*) of 459 nm and a quantum yield of 0.023 upon photoexcitation at *λ* = 430 nm (“SI”, Fig. S2). The PL spectrum is characterized with a vibronic spacing of 1040 cm^−1^. This spectral signature, together with the small Stokes shift of 1097 cm^−1^, collectively indicates that the π–π* transition state is responsible for the PL emission of DY1. The peak emission energy of 2.70 eV (corresponding wavelength: 459 nm) is close to the difference between the oxidation and reduction potentials (2.94 eV), indicating the central role of pyrazinoquinoxaline in the electronic behavior of DY1 (“SI”, Fig. S3).

**Figure 1:**
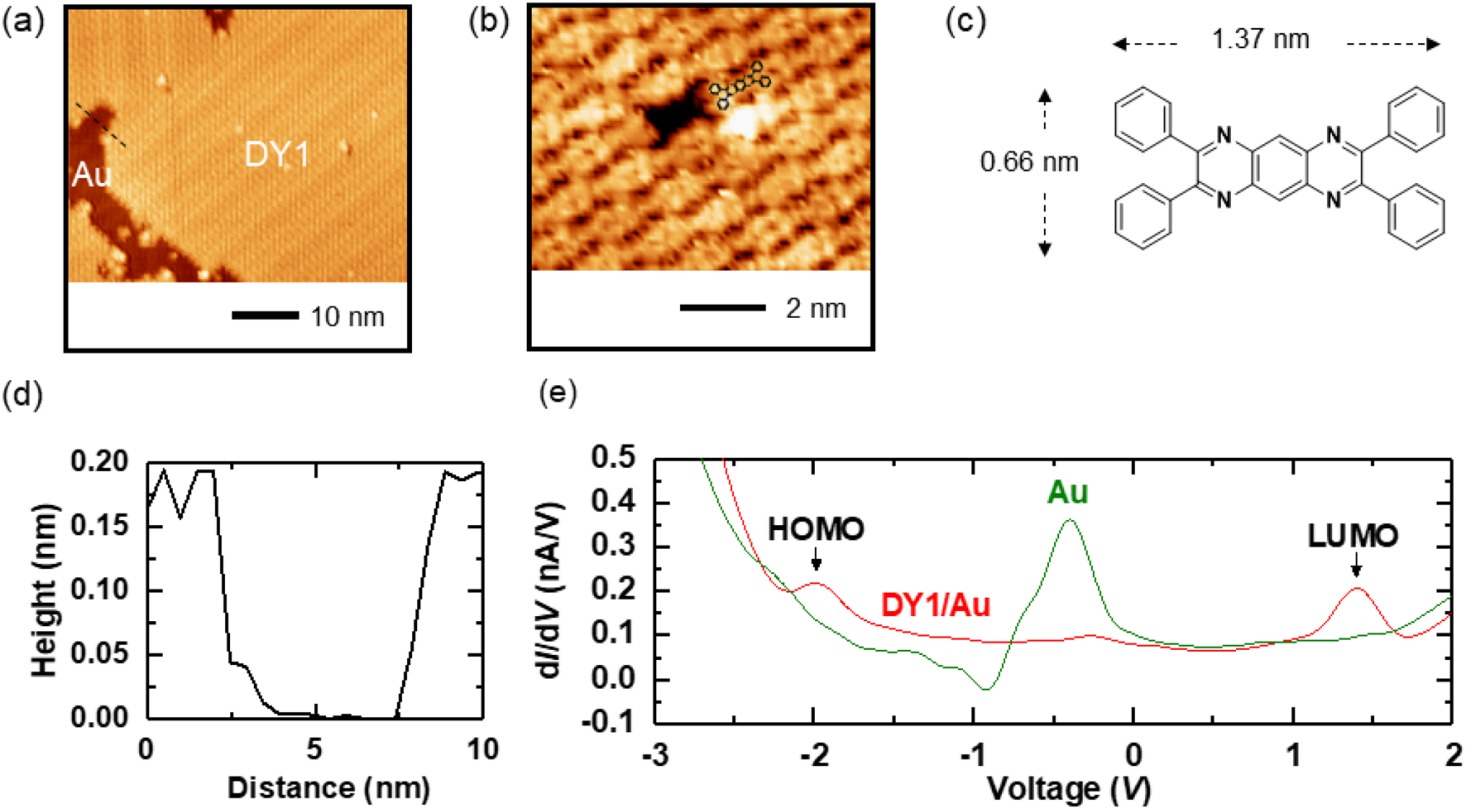
STM topographic images of monolayer DY1/Au(111) obtained at (**a**) *V* = 1 V and *I* = 20 pA and (**b**) *V* = 20 mV and *I* = 150 pA. The calculated molecular structure in (**c**) is inserted and superimposed near the single vacancy of the molecular layer. (**c**) DFT calculated structure of single DY1 molecule. (**d**) Height profile across the large molecular domain and the exposed Au surface (the black dashed line in (**a**)). (**e**) d*I*/d*V* spectroscopy results of DY1/Au(111) and Au(111) taken at *V* = 1.4 V, *I* = 100 pA and *V* = 1 V, *I* = 70 pA, respectively, at a temperature of ~ 10 K. Each result was averaged and smoothed from more than 30 scans.

Figure 1a,b show the representative STM topographic images of DY1 molecules grown atop the Au(111) surface. The DY1 molecules adsorb intact on the Au(111) surface and exhibit long-range ordered and close-packed domains. The height profile across the self-assembled area and the exposed Au surface (the black dashed line in Fig. 1a) shows that the thickness of the layer is only 0.2 nm, indicating the growth of monolayers (Fig. 1d). Interestingly, single-molecule vacancies in the molecular layer reveal topographic features that closely resemble the molecular structure of an individual DY1, which is calculated by density functional theory (DFT) (Fig. 1c and “SI”, Fig. S1). This identity suggests negligible intramolecular geometry change upon forming the molecular self-assemblies. A commensurate arrangement of organic molecules on metal surfaces has been reported in STM studies^14–16^. After depositing the molecules on the Au surface, the sample was kept at room temperature for several minutes before inserting the sample into the STM head (~ 10 K). It implies that the DY1 molecules can form self-assembled molecular structures with forming a closely packed zig-zag pattern (“SI”, Fig. S4) without further thermal treatment, where molecule–molecule interactions dominate molecule–substrate. In addition, the residual herringbone structure of the underlying Au(111) remains visible through the molecular layer, serving as a further proof that no chemisorption or surface reconstructions occur upon molecular adsorption.

We performed STS measurements of the self-assembled DY1 molecules, as shown in Fig. 1e. The differential tunneling conductance (d/d) is proportional to the density of states and directly reveals the electronic structure of the sample. The d/d spectra from DY1/Au(111) and Au(111) exhibit quite distinct features, as shown in Fig. 1e. The peaks near − 0.4 V in both regions originate from the surface state of Au(111)^17^. The two resonant peaks on DY1/Au(111), + 1.40 eV and − 1.98 eV, can be attributed to the LUMO and HOMO levels of DY1, respectively. The LUMO–HOMO gap (E_g_) of DY1 from dI/dV is estimated to be 3.38 eV, which is similar to the gap of an isolated DY1 molecule calculated quantum-chemically at the B3LYP level of theory (3.226 eV) (“SI”, Fig. S1). This indicates that the electronic structure of the DY1 molecules on the surface is almost preserved compared to that of free molecules; thus confirming that the molecules are very weakly bonded to the Au substrate. Based on the well-ordered intermolecular structure, almost unchanged LUMO–HOMO gap, and the lack of reconstruction of the Au herringbone structure, we conclude that the DY1 molecules prefer to be physisorbed on the Au substrate via van der Waals interactions.

The STM and STS results above show that well-ordered, self-assembled DY1 monolayers on Au can be obtained by a simple evaporation method and that the electronic structures of DY1 are not substantially perturbed on Au. At organic/metal interfaces, dipole layers can be formed due to several origins, including interfacial charge transfers, spatial redistribution of electron wave functions, and chemical reactions^6–12^. Such interfacial dipoles can cause a shift in the vacuum level (VL) of the organic material with respect to that of the metal. The magnitude of the VL shift, *Δ*, can be as large as 1 eV^6–9^. Negative (positive) *Δ* corresponds to the downward (upward) VL shift, and hence, the LUMO (HOMO) level moves close to the Fermi level of the metal electrode, *E*_F_^6–9^. As a result, a large magnitude *Δ* increases the hole/electron injection barrier from the organic to the metal. According to the STS data (Fig. 1e), the LUMO level of DY1 is at 1.40 eV above *E*_F,_ and its HOMO level is at 1.98 eV below *E*_F_. This suggests that no charge transfer across the DY1/Au interface occurs and that *Δ* should be negligibly small, indicating that DY1 is physisorbed on the Au^6–9^.

In addition to the Au(111) surface, experiments were also performed using Ag(100) to investigate the electrode material dependence of the interfacial electronic structure. The results show that DY1 molecules on the Ag(100) surface form a monolayer-thick layer with similar topographic features, as shown in the case of the Au substrate (“SI”, Fig. S5). However, d*I*/d*V* spectroscopy on the DY1/Ag system shows that the LUMO peak appears at 1.86 eV and that the HOMO peak is absent. This phenomenon points toward a higher degree of interaction between the molecules and the underlying Ag(100) substrate, compared to the DY1/Au(111) system. Stronger molecules/substrate interactions in the DY1/Ag system may induce broadening of the HOMO level^6–18^. On the other hand, the upshift of the LUMO level of DY1 on Ag may indicate electron transfer from the substrate to the molecule, considering the smaller electron affinity of Ag (1.30 eV) compared with that of Au (2.31 eV)^17^. Several analogous STS reports have shown significant modification of the electronic structures and *E*_g_ in some molecule/metal systems due to strong interactions^15–18^. It has been reported that a severe decrease in *E*_g_ can lead to charged and metallic molecular monolayers at the interface^18^.

**Figure 2:**
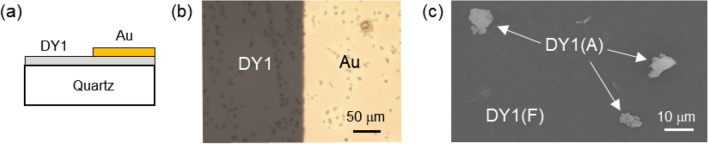
(**a**) Cross-sectional schematic diagram and (**b**) top-view optical microscope image of a DY1/quartz sample partially coated with a 60-nm Au thin film. (**c**) Aggregates with a lateral size of ~ 10 μm are clearly shown in a scanning electron microscopy image. The regions with and without such aggregates are indicated as DY1(A) and DY1(F), respectively.

DY1 molecules were evaporated on quartz substrates to enable optical characterizations of their assemblies. To study the influence of the Au layers, the DY1/quartz samples were partially coated with 60-nm-thick Au thin films using an electron-beam evaporator and a shadow mask, as illustrated in Fig. 2a. The Au thin film can be clearly distinguished from the bare DY1 region by optical microscopy (Fig. 2b). Dark spots in Fig. 2b correspond to aggregates of DY1 molecules. The typical shape and lateral size of the aggregate (~ 10 μm) can be examined by scanning electron microscopy (Fig. 2c). The height of the DY1 aggregate is estimated to be a few hundred nm from the atomic force microscopy (AFM) measurements (“SI”, Fig. S6a). Hereafter, the DY1 regions with and without such large aggregates will be denoted as ‘DY1(A)’ and ‘DY1(F)’, respectively. The formation of large aggregates of DY1 may be ascribed to the strong π–π interaction^4,5^. On the surface of DY1(F), bumps with ~ 20 nm height and ~ 300 nm lateral size were found from the AFM image (“SI”, Fig. S6b), also indicating strong intermolecular interactions.

**Figure 3:**
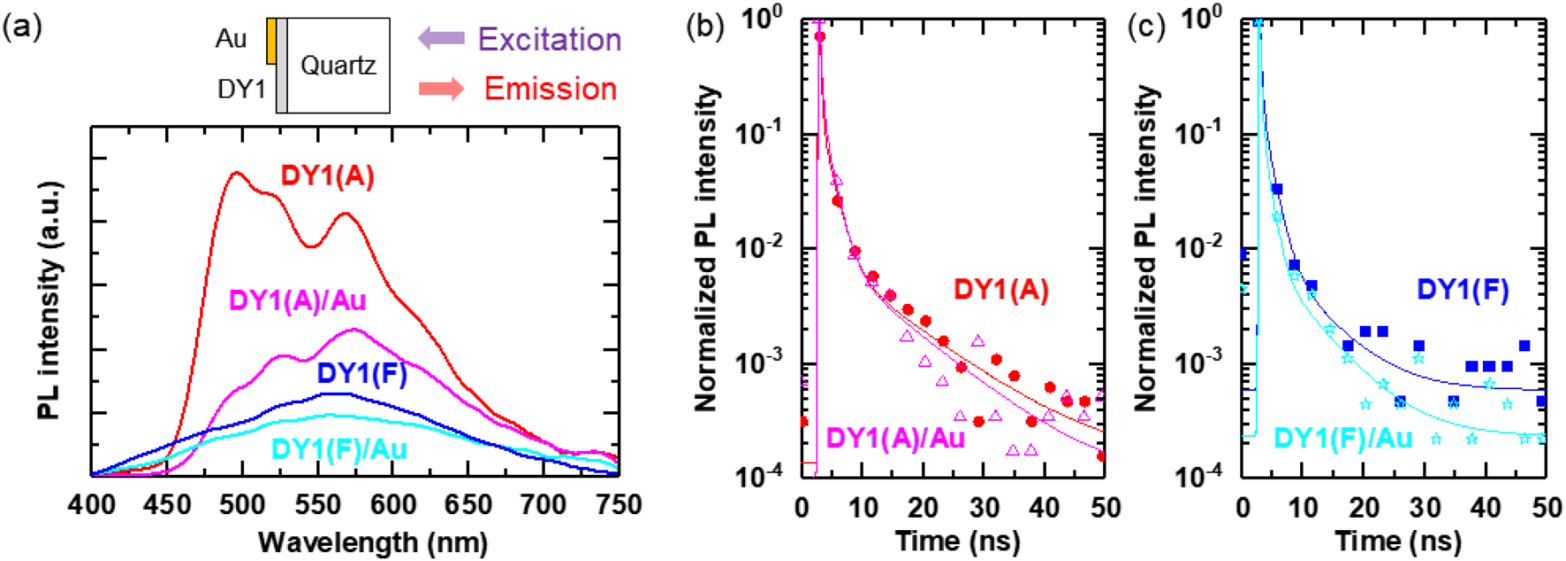
(**a**) Photoluminescence (PL) spectra of DY1(A), DY1(A)/Au, DY1(F), and DY1(F)/Au measured using a 375-nm excitation source with a beam diameter of ~ 1 μm. The measurement configuration is schematically illustrated above the spectra. (**b**, **c**) The time-resolved fluorescence decay curve of each region obtained from the fluorescence lifetime imaging microscopy (FLIM) images. Symbols and solid lines correspond to the raw data and least-square fitting, respectively. A 405-nm laser with a beam diameter of ~ 660 nm was used for the FLIM studies.

PL spectra of the DY1 assemblies/quartz samples were obtained under excitation at *λ* = 375 nm (Fig. 3a), of which the energy is comparable to *E*_g_ of DY1 (3.38 eV) estimated from the STS data (Fig. 1c). The PL emission of the evaporated films of DY1 contains a multitude of bands that cover the entire visible region. The broad-band emission suggests that intermolecular species, such as aggregates, contribute to PL emission. The PL spectrum of DY1(A) contains the major peak at 500 nm and the minor peak at 570 nm. Both bands retain the vibronic structures and are bathochromically shifted from the monomeric emission (459 nm) of DY1 (“SI”, Fig. S2). These spectral behaviors exclude excimers as being responsible for the emission and suggest the presence of DY1 assemblies with varied stacking geometries. Notably, DY1(F) exhibits broad emission with a peak at *λ* = 570 nm. The relative decrease in the 500 nm emission of DY1(F) indicates the harvest of DY1 excitons by the low-energy assemblies.

The Au thin film can work as a mirror to reflect incident and emitted light, which can increase the PL intensity from the Au-coated DY1. However, the PL intensity from the Au-coated region is smaller than that from the bare DY1 region (Fig. 3a). This suggests that the Au layer suppresses radiative decay in DY1. It can also be noted that the Au thin film modifies the relative intensity ratio of the major and minor bands in DY1(A), as shown in Fig. 3a and Fig. S7. If the major-band-relevant vibronic level is severely modified near the Au layer, then the major-band emission in the PL spectra can be reduced.

Figure 3b,c shows that the Au thin film decreases the fluorescence lifetime of DY1(A) and DY1(F). The decreased lifetime by the Au layer is also clearly shown in the fluorescence lifetime imaging experiments (“SI”, Fig. S8a,b). The weighted lifetimes of DY1(F)/Au, DY1(F), DY1(A)/Au, and DY1(A) are estimated to be 1.2, 1.5, 1.7, and 2.3 ns, respectively (Fig. S8c). The interfacial charge transfer can suppress the radiative recombination of photogenerated excitons, leading to a decrease in the lifetime and PL intensity^23^. In addition, nonradiative energy transfer from excitons near the Au surface to surface plasmons can reduce the lifetime of the fluorescence emission^24^.

**Figure 4:**
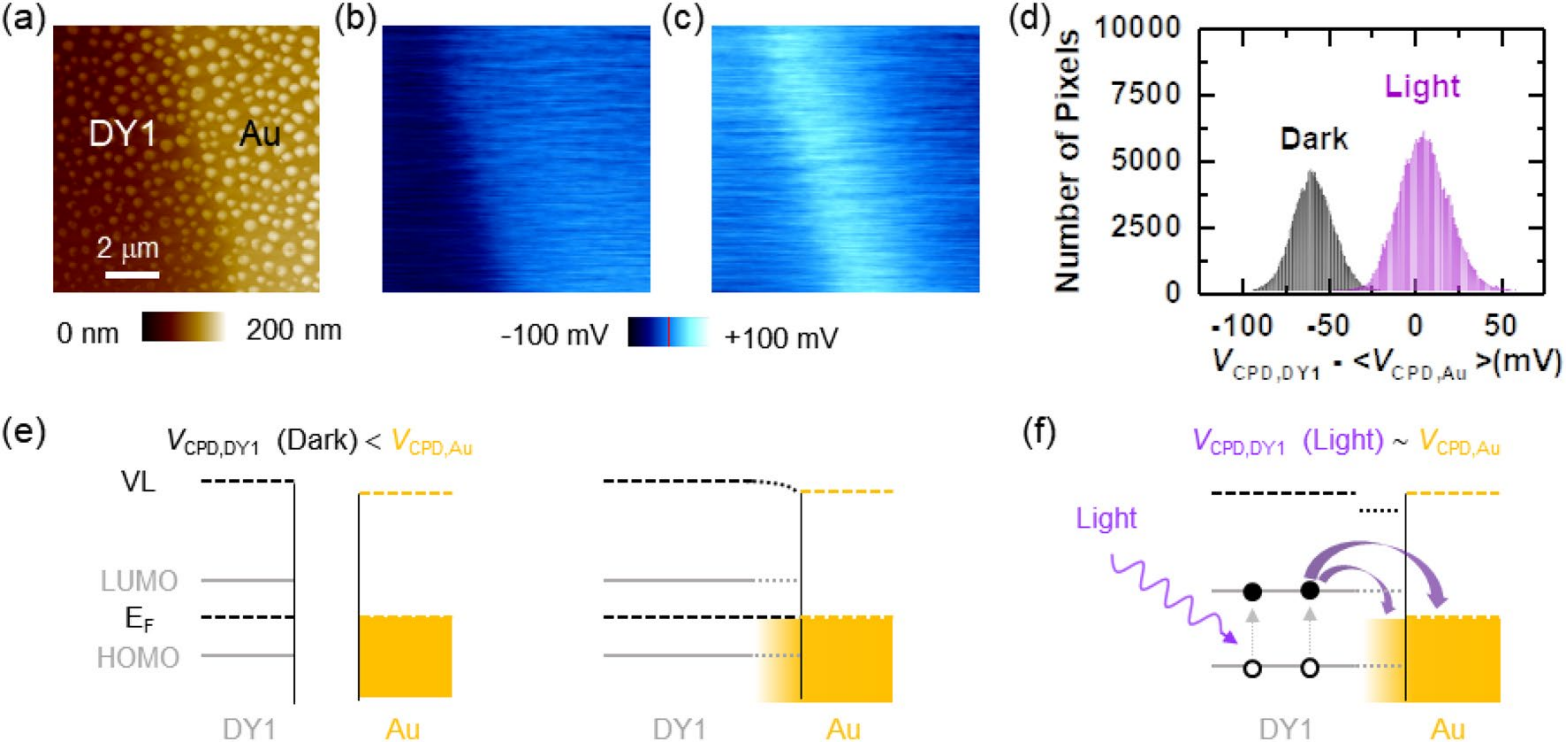
(**a**) AFM topographic image of DY1 and Au. *V*_CPD,DY1_ − <V_CPD,Au_> maps (**b**) in the dark and (**c**) under 405-nm light illumination at 8.28 mW/cm^2^. <V_CPD,Au_> corresponds to the average *V*_CPD_ measured on the Au thin film. (**d**) Histogram of the number of pixels in the dark and light *V*_CPD_ data. Proposed energy level alignments of (**e**) DY1 and Au before and after contact in the dark and (**f**) the DY1/Au interface under 405-nm illumination. A discontinuous Au layer can be present near the DY1-Au boundary, which is illustrated in (**e**) and (**f**). The filled and open circles in (**f**) represent electrons and holes generated by incident light.

Kelvin probe force microscopy (KPFM) enables measurements of the contact potential difference (*V*_CPD_) and *V*_CPD_ ≡ [WF_tip_ − WF_sample_]/*e*, where WF_tip_ and WF_sample_ are the work functions (WFs) of the KPFM tip and the sample, respectively, and *e* is the electron charge^25,26^. Figure 4a shows the surface morphology of the DY1(F) region partially coated with the Au thin film. The topographic image shows bumps on the surface, of which the typical height and lateral size are 10 nm and 2–300 nm, respectively. This suggests that the strong intermolecular interaction in DY1 leads to the generation of aggregates, as discussed above^4,5^. The height difference of the bare and Au-coated regions confirms the thickness of the Au thin film (60 nm).

The *V*_CPD_ maps measured on DY1 and Au are very uniform without topographic artifacts, as shown in Fig. 4b. The *V*_CPD_ at DY1 (*V*_CPD,DY1_) is smaller than that of Au (*V*_CPD,Au_) by 60 meV, and the WF of our DY1 layer should be slightly larger than that of ambient-exposed Au (4.5 ± 0.1 eV)^9^. Figure 4c shows that illumination with 405-nm light increases *V*_CPD,DY1_. *V*_CPD,Au_ should be kept constant since the Au layer was grounded. Thus, [*V*_CPD,DY1_ − *V*_CPD,Au_] clearly reveals a light-induced increase in *V*_CPD,DY1_ (Fig. 4d). *V*_CPD,DY1_ returns to the initial dark state value after turning off the light source, and hence, the reversible *V*_CPD_ change should originate from the photogeneration of charge carriers.

Figure 4e shows a schematic diagram of the energy levels of DY1 and Au before (left) and after (right) contact in the dark. Near the DY1-Au boundary, a Au layer with gradually varying thickness can be present due to incomplete blocking of the shadow mask. In the corresponding region, the *V*_CPD_ continuously changes, as observed in Fig. 4b. The relative positions of HOMO and LUMO with respect to *E*_F_ could be determined from the STS spectra (Fig. 1e): the LUMO and HOMO levels of DY1 are located at 1.40 eV above and 1.98 eV below *E*_F_, respectively. The WF (i.e., the separation between VL and the Fermi level) values of DY1 and Au were obtained from the KPFM-measured *V*_CPD_ data. As discussed above, the VL shift at the DY1/Au interface, *Δ*, is supposed to be very small. Thus, the so-called vacuum level alignment is assumed in the energy level alignment at the DY1/Au contact^6–9^.

Notably, 405-nm light, with a photon energy larger than the *E*_g_ of DY1, can generate excitons in DY1. Such excited state decay occurs not only because of recombination in DY1 but also charge separation at the DY1/Au interface^24,27^. Excited-state DY1 can either donate electrons in its higher singly occupied molecular orbital (HSOMO) to Au or accept electrons from Au to its lower singly occupied molecular orbital (LSOMO). The former (latter) process can occur when the *E*_F_ of Au is below (above) the HSOMO (LSOMO) of DY1^27^. The energy level alignment at DY1/Au (Fig. 4e) suggests that electron transfer processes from DY1 to Au and vice versa are possible. The light-induced increase in *V*_CPD,DY1_ indicates positive charging of DY1^19,25,26^. This suggests that the electron transfer from DY1 to Au is dominant and that the remaining holes in DY1 increase *V*_CPD,DY1_. The net charge in the DY1 region suppresses the separation of photogenerated excitons and drift of electrons toward the Au region. Approximately 10 min was spent taking one *V*_CPD_ map, and the measured *V*_CPD_ signals represent the saturated states under illumination^28^. The light-induced charge separation at DY1/Au can explain the decrease in intensity and decay time of the PL (Fig. 3a–c).

It should be noted that the light-induced *V*_CPD_ change is the largest at the DY1-Au boundary. The region showing the clear *V*_CPD_ contrast (Fig. 4b) is somewhat shifted to the left from the region showing a height change (Fig. 4a). This can be attributed to the gradual thickness variation of the Au thin film prepared by a shadow mask. As the thickness decreases, discontinuous islands should appear instead of a continuous film^29^. Exciton separation and subsequent electron transfer from DY1 to such Au islands can occur. Zhang et al. reported that negative charging of Au nanoparticles could increase the measured *V*_CPD_^25^. This charge-induced *V*_CPD_ change could be as large as 0.6 V, depending on the amount of charge and particle size. Charged Au islands help dissociate the photogenerated excitons and drift the charge carriers near the DY1-Au boundary region. The surface plasmon resonance of the Au thin film can appear in discontinuous island-like forms with sub-10-nm thickness^30^. The expected resonance wavelength ranges from 550 to 750 nm, which is much longer than that of our illumination system (405 nm). Therefore, plasmonic effects at the DY1-Au boundary may not significantly contribute to the *V*_CPD_ change in our experiments.

## Conclusion

Fluorescent π-conjugated molecules of DY1 were evaporated on Au(111) and quartz substrates at room temperature. The formation of uniform DY1 monolayers with well-ordered herringbone patterns could be visualized from low-temperature (10 K) scanning tunneling microscopy images. The tunneling conductance spectra and contact potential difference data revealed a DY1 gap of 3.38 eV and energy level alignment at the DY1/Au interface. The estimated gap of the self-assembled layer agreed well with that of an isolated molecule obtained from theoretical calculations. The electronic structures derived from all these results suggested that the self-assembled molecules were physisorbed on the Au surface without either integer charge transfers or chemical reactions at the interface. Both bare DY1 and Au-coated DY1 on quartz exhibited broadband photoluminescence in the visible wavelength range: the emission lifetime at the DY1/Au interface was 70–80% of that of bare DY1. The comparison of surface potential maps in the dark and under illumination suggested that separation of photogenerated excitons and electron transfer from DY1 to Au should occur. This could explain the decreased PL lifetime at the DY1/Au interface.

## Methods

### Syntheses of DY1

DY1 was prepared following the method reported previously (Ref. 5 - Adv. Optical Mater. 2019, 7, 1900201). Yellow powder; ^1^H NMR (300 MHz, CD_2_Cl_2_) *δ* (ppm) 8.95 (s, 2H), 7.62 (m, 8H), 7.42 (m, 12H); ^13^C{^1^H} NMR (126 MHz, CD_2_Cl_2_) *δ* (ppm) 155.8, 140.9, 139.5, 130.5, 129.8, 129.1, 128.7. HR MS (FAB^+^, m-NBA): Calcd for C_34_H_23_N_4_ ([M + H]^+^), 487.1917; found, 487.1927.

### Quantum chemical calculations

Geometry optimization was performed using Becke’s three-parameter (B3LYP) exchange–correlation functional and the 6 − 311 + G(d,p) basis set. Frequency calculations were subsequently performed to assess the stability of the convergence. The length and width of DY1 were estimated from the optimized geometry. Time-dependent density functional theory (TD-DFT) calculations were carried out for the optimized geometries using the same functional and basis sets. Thirty states were considered for the TD-DFT calculations. Geometry optimization and single-point calculations were performed using the Gaussian 09 program^31^.

### Steady-state UV–Vis absorption measurements of solutions

UV–Vis absorption spectra were collected on a spectrophotometer (Cary 300, Agilent) at 298 K. Sample solutions were prepared prior to measurements at a concentration of 10 μM in toluene, unless otherwise stated. The solution was placed in a quartz cell (Hellma, beam path length = 1.0 cm).

### Steady-state PL measurements of solutions

PL spectra were obtained using a Photon Technology International Quanta Master 400 scanning spectrofluorometer at 298 K. The solutions used for the steady-state UV–Vis absorption studies were employed for the PL measurements. The solutions were deaerated by bubbling with Ar prior to the measurements. A quartz cell (Hellma, beam path length = 1.0 cm) was employed.

### Electrochemical characterization

Cyclic and differential pulse voltammetry experiments were carried out using a CH Instruments CHI630B potentiostat using a three-electrode cell assembly. A Pt wire and a Pt microdisk were used as the counter and working electrodes, respectively. A Ag/AgNO_3_ couple was used as a pseudoreference electrode. Measurements were carried out in Ar-saturated acetonitrile (2.0 mL) using 0.10 M TBAPF_6_ as the supporting electrolyte at scan rates of 100 mV s^−1^ (cyclic voltammetry) and 4.0 mV s^−1^ (differential pulse voltammetry). A ferrocenium/ferrocene couple was employed as the external reference.

### Evaporation of DY1

DY1 molecules were thermally evaporated at a growth rate of 0.005 nm min^−1^ onto precleaned Au(111) and Ag(100) surfaces prepared by repeated Ar gas sputtering and annealing at 500 °C. DY1 layers were also prepared on quartz substrates with a deposition rate of 0.1 nm min^−1^ for 20 min. The expected thickness of the molecular layer atop the quartz substrate was 2 nm. Evaporation was performed in a vacuum chamber with a base pressure of 10^–8^ mbar, and the pressure during evaporation remained below 10^–7^ mbar to avoid any contamination. The thickness of the deposited molecular layer was measured using a quartz thickness monitor and calibrated using STM topographies.

### STM and STS measurements

All STM and STS measurements were performed using a commercial UHV LT-STM (RHK Technology), which was operated at a temperature of 10 K in a UHV environment (< 1 × 10^–10^ mbar) for negligible contamination and thermal drift. The Pt/Ir tip was prepared using controlled contact with the sample, and a bias voltage was applied to the sample. STM topographic images were obtained by enabling STM feedback and recording voltage applied to the z-piezo with a constant tunneling current. d/d spectra were measured at a specific region by disabling the STM feedback and recording the tunneling current as a function of the bias voltage. To enhance the signal-to-noise ratio, a small modulation voltage of 10 mV_pp_ at 780 Hz was added to the bias voltage, and the differential conductance of d*I*/d*V* was acquired using a lock-in amplifier.

### KPFM measurements

*V*_CPD_ and the surface morphology of the sample were measured using a KPFM system (XE-100, Park Systems) in a nitrogen-purged glove box with Pt/Ir tips (NSG01Pt, NT-MDT). The dark states were characterized after storing the sample for more than 3 h in a light-blocked glove box. A laser diode with a wavelength of 405 nm and power density of 8 mW cm^−2^ was used as a light source to examine the illumination-induced *V*_CPD_ changes.

### Optical characterization of the evaporated samples

Steady-state PL spectra from a selected region were obtained using a confocal microscope (MicroTime-200, Picoquant) with a 20× objective lens (beam diameter: ~ 1 μm), a 375-nm diode laser excitation source, and a spectrophotometer (F-7000, Hitachi). The fluorescence decay time studies were carried out using an inverted-type scanning confocal microscope (SP8 FALCON, Leica Microsystems) with a 20× objective lens (beam diameter: ~ 660 nm), a picosecond 405-nm laser source, and a hybrid photon detector. FLIM images, consisting of 512 × 512 pixels, were recorded using a galvo-stage and time-correlated single-photon counting technique. All data manipulation for the obtained fluorescence decays were applied using Leica suite software (LAS X Ver.3.5.2).

## Supplementary Information

Supplementary Information.
