# Supplementary material for: Electronic structures and optical characteristics of fluorescent pyrazinoquinoxaline assemblies and Au interfaces
Source: Sci Rep. 2021 Aug 20;11:16978. doi: 10.1038/s41598-021-96437-x (PMC8379188; doi:10.1038/s41598-021-96437-x)
Supplement: Supplementary file 1 — Supplementary Information. [file 41598_2021_96437_MOESM1_ESM.pdf]

Supplementary Information

Electronic Structures and Optical Characteristics of Fluorescent Pyrazinoquinoxaline Assemblies and Au Interfaces

Soyeong Kwon,[1] Dong Yeun Jeong,[2] Weon-Sik Chae,[3] Kyungju Noh,[1,4] P. Devi,[4,5] Luciano Colazzo,[4,5] Youngmin You,[2,*] Taeyoung Choi,[1,*] and Dong-Wook Kim[1,*]

[1] Department of Physics, Ewha Womans University, Seoul 03760, Korea

[2] Division of Chemical Engineering and Materials Science, and Graduate Program in System Health Science and Engineering, Ewha Womans University, Seoul 03760, Korea

[3] Daegu Center, Korea Basic Science Institute (KBSI), Daegu 41566, Korea

[4] Center for Quantum Nanoscience, Institute for Basic Science (IBS), Seoul 03760, Korea

[5] Ewha Womans University, Seoul 03760, Korea

* Correspondence and requests for materials should be addressed to Y.Y. (email: odds2@ewha.ac.kr), T.C. (email: tchoi@ewha.ac.kr), and D.-W.K. (email: dwkim@ewha.ac.kr)

Table S1. Report of the optimized geometry of radical anion of DY1.

atom	Cartesian coordinate			atom	Cartesian coordinate		
C	-3.54024000	0.73372300	-0.08262100	H	5.73012800	0.32448000	1.52915800
C	-1.20498400	0.69374500	-0.22101300	C	5.88184300	3.65387100	-0.45969100
C	-1.20492000	-0.69369400	0.22100700	H	3.97546100	3.07859700	-1.27682400
C	-3.54017700	-0.73360500	0.08300300	C	6.92077400	3.30095300	0.41592900
C	-0.00008400	1.35944100	-0.45441600	H	7.64654100	1.83828300	1.82174600
C	0.00009000	-1.35943900	0.45437000	H	5.92983500	4.58522000	-1.00578500
C	1.20499000	-0.69374400	0.22096500	H	7.76450900	3.96390300	0.54760000
C	1.20492700	0.69369500	-0.22105400	C	-4.73046000	1.57721600	0.06702300
C	3.54018400	0.73360800	-0.08304600	C	-4.79134600	2.81097900	-0.62253700
C	3.54024600	-0.73372100	0.08257000	C	-5.78059400	1.23706900	0.95517800
H	0.00008900	-2.39564700	0.75444000	C	-5.88282300	3.65317200	-0.46036700
N	-2.39350900	-1.36967300	0.31281800	H	-3.97643200	3.07800400	-1.27758400
N	-2.39366000	1.36979800	-0.31252600	C	-6.85982900	2.09721800	1.12829700
N	2.39351500	1.36967700	-0.31285700	H	-5.72977000	0.32486400	1.53013200
N	2.39366700	-1.36979600	0.31247900	C	-6.92148500	3.30043300	0.41563300
C	4.73046700	-1.57721600	-0.06705400	H	-5.93115900	4.58423800	-1.00691600
C	5.78060600	-1.23708900	-0.95521100	H	-7.64656700	1.83828100	1.82232400
C	4.79134500	-2.81097000	0.62252400	H	-7.76537600	3.96322000	0.54712800
C	6.85983900	-2.09724500	-1.12831000	C	-4.73017200	-1.57729000	-0.06685400
H	5.72978700	-0.32489700	-1.53018400	C	-4.79055200	-2.81146500	0.62209200
C	5.88281900	-3.65317000	0.46037500	C	-5.78061800	-1.23698800	-0.95462200
H	3.97642600	-3.07798000	1.27757200	C	-5.88183600	-3.65385200	0.45977400
C	6.92148700	-3.30044900	-0.41562500	H	-3.97542600	-3.07857000	1.27683700
H	7.64658000	-1.83832500	-1.82233900	C	-6.85963500	-2.09734600	-1.12791500
H	5.93114800	-4.58422700	1.00693900	H	-5.73017000	-0.32449700	-1.52914000
H	7.76537500	-3.96324200	-0.54710500	C	-6.92079300	-3.30094500	-0.41581900
C	4.73017000	1.57729600	0.06686400	H	-5.92981400	-4.58519000	1.00588600
C	5.78059000	1.23698200	0.95465900	H	-7.64659900	-1.83829800	-1.82164100
C	4.79056700	2.81148400	-0.62205800	H	-7.76453500	-3.96389400	-0.54745300
C	6.85959800	2.09734100	1.12800100	H	-0.00008200	2.39564900	-0.75448100

Table S2. Electronic transitions of DY1.

state	energy (eV)	participating molecular orbitals (expansion coefficient)	oscillator strength (au)
T_1	1.77	HOMO–1 → LUMO (0.67)	-
T_2	2.27	HOMO → LUMO (0.68)	-
T_3	2.72	HOMO–5 → LUMO (0.60)	-
S_1	2.74	HOMO → LUMO (0.70)	0.9562

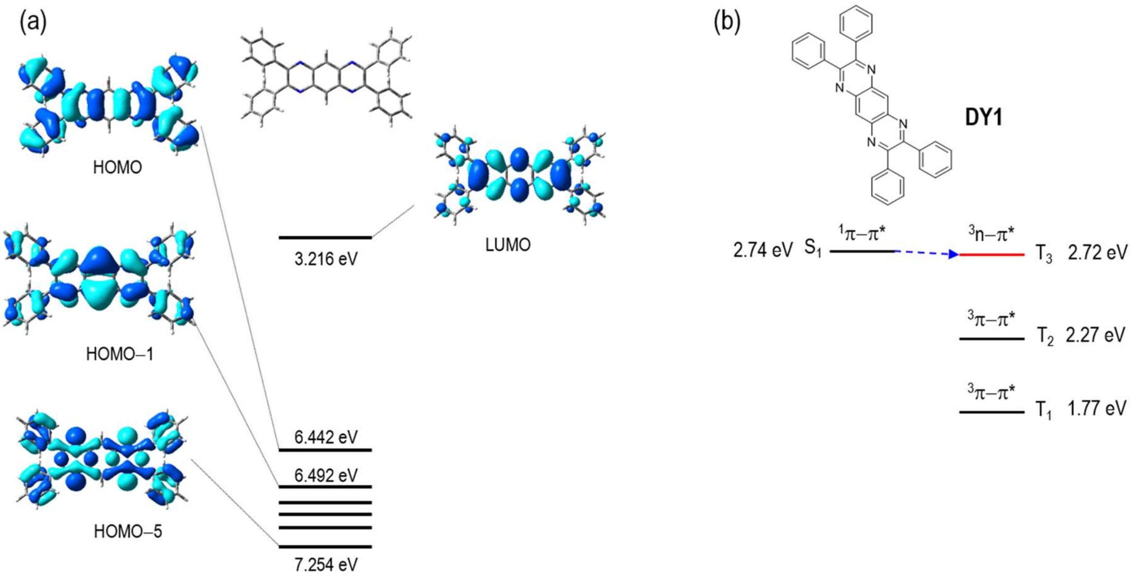

Figure S1. (a) Simulated (TD-B3LYP/6-311+g(d,p); 30 states) isosurfaces of the molecular orbitals that participate in the lowest electronic transitions in DY1. (b) The lowest electronic transition states of DY1.

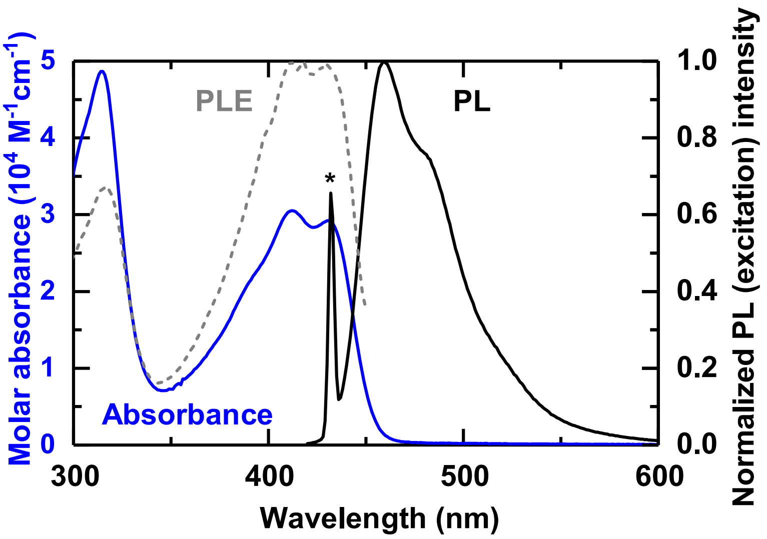

Figure S2. Photoluminescence (black solid line; $\lambda_{excitation}$ = 430 nm) and photoluminescence excitation (gray dashed line; $\lambda_{emission}$ = 459 nm) spectra, and UV–vis absorption spectrum (blue solid line) for 10 μM DY1 recorded in Ar-saturated toluene. The peak marked with an asterisk corresponds to the excitation beam.

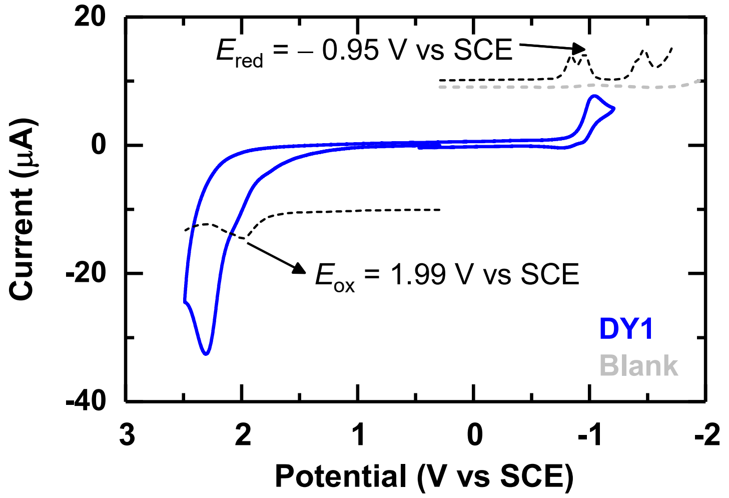

Figure S3. Cyclic (blue solid lines) and differential pulse (black dotted lines) voltammograms of 2.0 mM **DY1** in Ar-saturated acetonitrile containing 0.10 M tetrabutylammonium hexafluorophosphate. A Pt disk and a Pt wire for the working and counter electrodes, respectively; an $Ag/AgNO_3$ pseudo reference electrode. Scan rate = 0.1 V s^{-1} (cyclic voltammetry) and 0.004 V s^{-1} (differential pulse voltammetry).

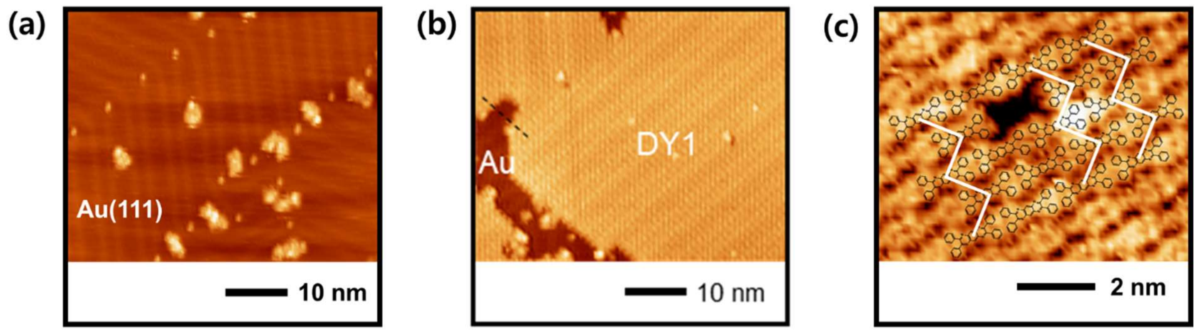

Figure S4. STM topographic images of DY1/Au(111). (a) DY1 coverage of <0.005 ML, which represents unperturbed Au(111) surface (*V* = 1 V and *I* = 50 pA), (b) DY1 coverage of ~1 ML (*V* = 1 V and *I* = 20 pA); The distances of the nearest neighboring herringbone structure for (a) and (b) are 2.50$\pm$0.06 nm and 2.53$\pm$0.06 nm respectively. This shows that the Au (111) crystal structure remains unperturbed upon DY1 adsorption, and (c) zoomed-in STM image of 1ML DY1 on Au(111); The calculated DY1 molecular structures are overlaid based on the topographic STM image and single molecular defect. The molecules are adsorbed onto the surface with a planar geometry and self-assembled with forming a closely packed zig-zag pattern.

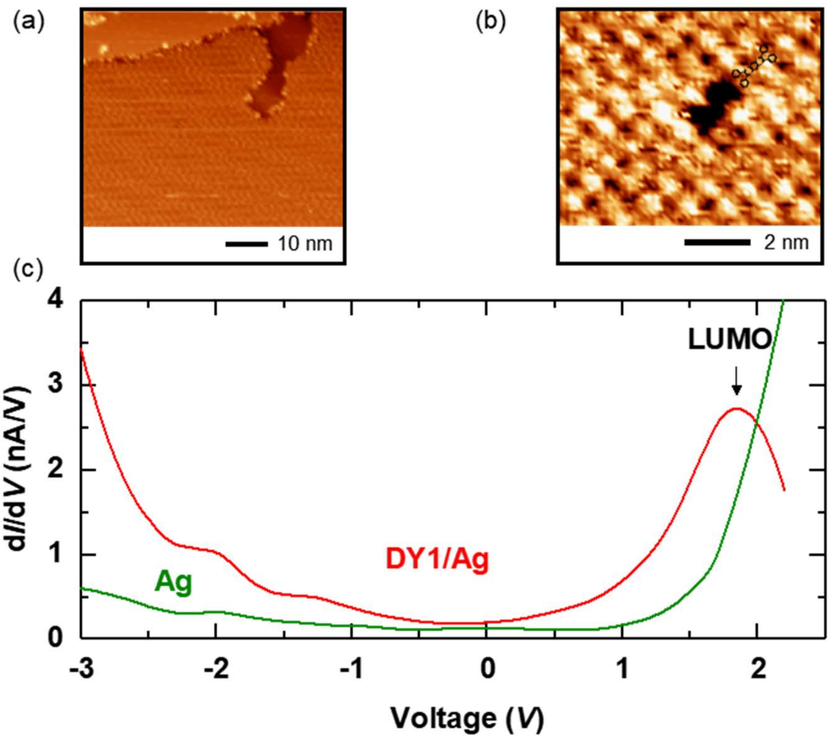

Figure S5. STM topographic images of DY1/Ag(100) obtained at (a) V = 0.5 V and I = 20 pA and (b) V = 0.5 V and I = 60 pA. (c) dI/dV spectroscopy results of DY1/Ag(100) and Ag(100) taken at V = 0.5 V, I = 60 pA at the temperature of ~10 K. Each result was averaged and smoothed from more than 5 scans.

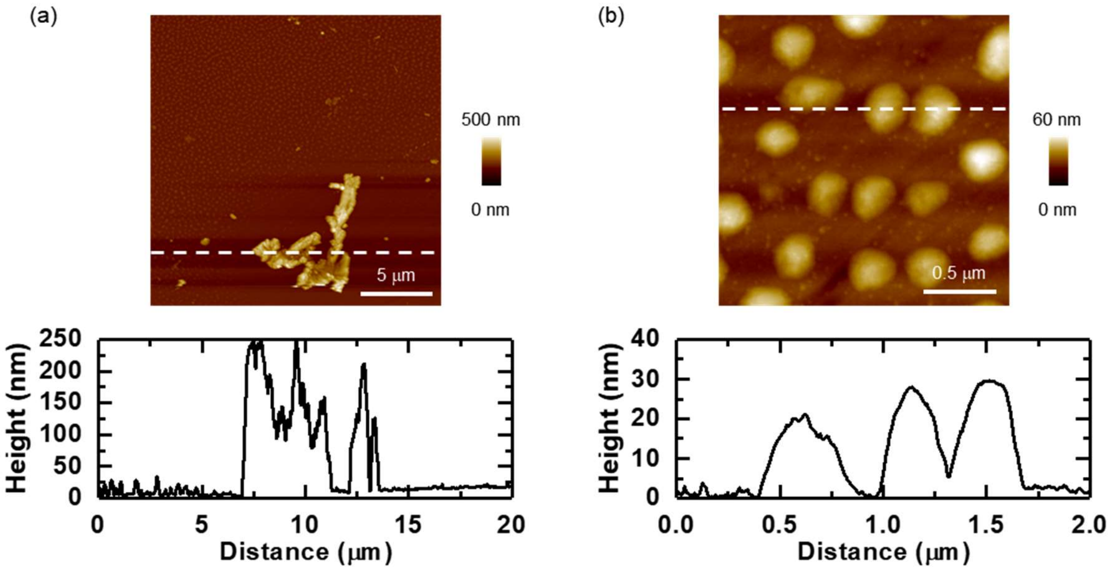

Figure S6. Typical AFM images of a DY1/quartz sample in (a) DY1(A) and (b) DY1(F). The vertical and lateral sizes of the aggregates and bumps can be estimated from the height profiles across the dashed lines.

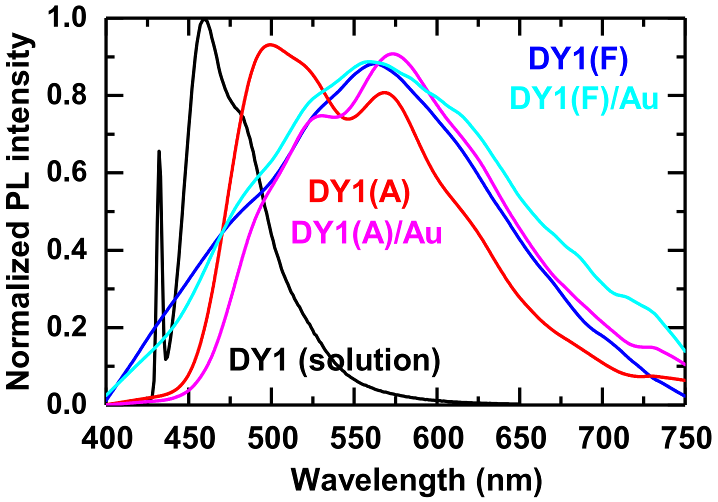

Figure S7. Normalized PL spectra of DY1(A), DY1(A)/Au, DY1(F), and DY1(F)/Au, and 10 μM DY1 in Ar-saturated toluene. The excitation wavelengths for the solid film and the solution forms were 375 nm and 430 nm, respectively.

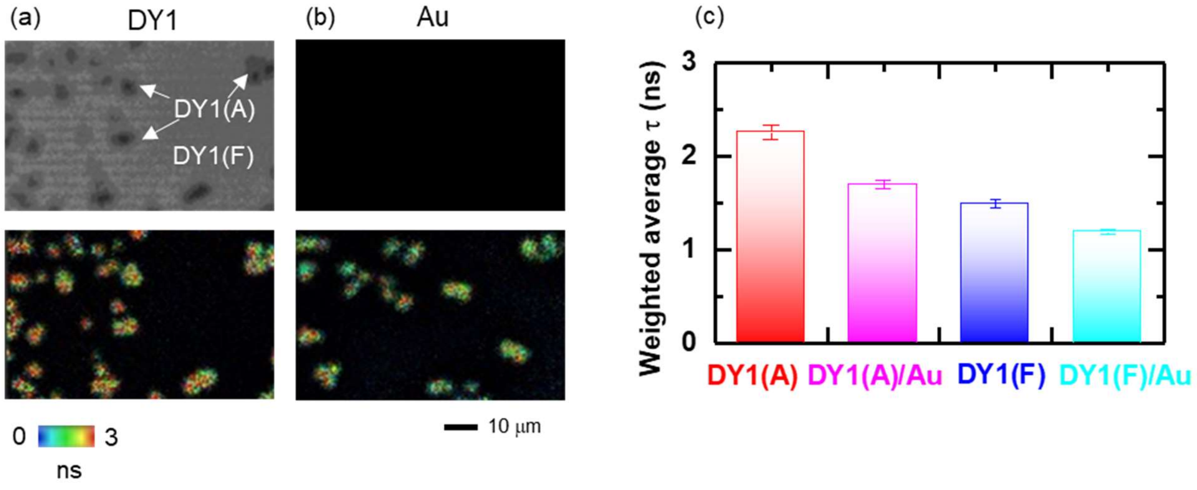

Figure S8. Transmission-mode bright-field images and fluorescence lifetime imaging (FLIM) images of DY1/quartz samples (a) without and (b) with the Au thin film under 405-nm light illumination. In (a), the regions with and without such aggregates are indicated as DY1(A) and DY1(F), respectively. The emitted light in the wavelength range of 450~750 nm was collected. (c) Weighted average lifetime of DY1(A), DY1(A)/Au, DY1(F), and DY1(F)/Au.
